# Effect of Prenatal Perineal Massage on Postpartum Perineal Injury and Postpartum Complications: A Meta-Analysis

**DOI:** 10.1155/2022/3315638

**Published:** 2022-07-14

**Authors:** Qiuxia Chen, Xiaocui Qiu, Aizhen Fu, Yanmei Han

**Affiliations:** ^1^Department Obstetrics, Hainan Women and Children's Medical Center, Haikou, 570216 Hainan, China; ^2^Department of Medical Genetics, Haikou Maternal and Child Health Hospital, Haikou, 570203 Hainan, China; ^3^Department Obstetrics, Haikou Maternal and Child Health Hospital, Haikou, 570203 Hainan, China

## Abstract

**Background:**

The efficacy of perineal massage is controversial. The study was aimed at comparing the effects of perineal massage on perineal injury and complications.

**Methods:**

PubMed, Embase, the Cochrane Library, and ISI Web of Science were searched for literature on the relationship between prenatal perineal massage and postpartum perineal injury and complications until April 2022. Indicators included postpartum perineal tears, perineotomy, postpartum perineal pain, natural labour, and postpartum incontinence. Finally, RevMan5.4 software was used to analyze the extracted data.

**Results:**

A total of 6487 subjects in 16 studies were included, with 3211 who received perineal massage and 3276 did not. There was no significant difference in 1-2 degree perineal tearing between the intervention group and the control group (RR = 0.96, 95% CI [0.90, 1.03], *P* = 0.30), and there was no heterogeneity between studies (*P* = 0.62, *I*^2^ = 0%), indicating publication bias. Compared with the control group, prenatal perineal massage significantly reduced the incidence of 3-4 degree perineal tears (RR = 0.56, 95% CI [0.47, 0.67], *P* < 0.00001), and there was no heterogeneity between studies (*P* = 0.16, *I*^2^ = 30%), indicating publication bias. Compared with the control group, prenatal perineal massage reduced the risk of lateral perineal resection (RR = 0.87, 95% CI [0.80, 0.95], *P* = 0.001), and there was no heterogeneity between studies (*P* = 0.14, *I*^2^ = 31%), and there was no publication bias. Compared with the control group, prenatal perineal massage reduced the risk of postpartum pain at 3 months (RR = 0.64, 95% CI [0.51, 0.81], *P* = 0.0002). There was no significant heterogeneity among studies (*P* = 0.23, *I*^2^ = 31%).

**Conclusion:**

Compared with no prenatal perineal massage, prenatal perineal massage can reduce the risk of perineal injury, the incidence of lateral perineal resection, and the incidence of long-term pain.

## 1. Introduction

Perineal injury, which refers to the injury that occurs in the genital area associated with laceration during delivery, has a high incidence of 30-85% in vaginal delivery [1]. It can cause perineal pain, difficulty in sexual intercourse, urinary incontinence, and other complications that greatly impact the physical and mental health of pregnant women. Although perineum incision is often offered preemptively to avoid perineum injury, the evidence supporting its efficacy remains elusive. Moreover, the utility of perineal incision is also limited by associated complications and psychologically decreases a woman's sexual desire and esteem. Currently, routine perineum incision is no longer recommended.

Perineal massage is a well-known treatment modality that has been shown [2] to stimulate nerve endings in the skin, enhance perineal blood circulation, improve the elasticity and ductility of perineal tissue, broaden the vaginal opening, reduce the probability of perineal incision, and reduce perineal tear. In addition, it facilitates vaginal delivery and probably reduces the risk of perineal injury by stimulating the child's head during childbirth. Currently, studies [3, 4] about the effect of prenatal perineal massage on the incidence of perineal tears and episiotomy reported inconsistent results. For instance, Ibrahim [5] reported that prenatal perineal massage did not benefit the mother more than Kegel exercises. The efficacy of antenatal perineal massage is controversial. To further explore the impact of prenatal perineal massage on postpartum perineal injury and postpartum complications, we conducted this systematic review and meta-analysis to update the available evidence to determine whether prenatal perineal massage can reduce the risk of perineal trauma and postpartum complications.

## 2. Materials and Methods

### 2.1. Literature Search

PubMed, Embase, the Cochrane Library, ISI Web of Science, and other databases were searched. The search time was set from its establishment to April 2022. Articles and studies about the impact of prenatal perineal massage on postpartum perineal injury and postpartum complications were collected. The search terms were “Antenatal perineal massage”, “Perineal trauma”, “Episiotomy”, and other similar phrases. The joint search was carried out with subject words and free words. References to the target literature were also examined.

### 2.2. Inclusion and Exclusion Criteria

Inclusion criteria were as follows: (1) study type: randomized controlled studies (RCTs); (2) participants: primipara or postmenopausal women undergoing prenatal care; (3) intervention group: prenatal perineal massage at 34-36 weeks of pregnancy; (4) control group: no perineal massage before delivery; and (5) results: the main results included the risk of perineal tear, the incidence of perineal incision, and natural vaginal delivery. Secondary outcomes were perineal pain (assessed by visual analogue scale (VAS)), urinary incontinence, and fecal incontinence at 3 months postpartum. Exclusion criteria were as follows: (1) nonrandomized trials, in vitro study, or animal study; (2) study overlap; (3) literature with incomplete data or no research indicators; and (4) unrelated studies.

### 2.3. Data Extraction and Quantitative Evaluation

Two researchers screened the data from the included literature. Controversies emerged were solved through discussion or consultation the third researcher. The extracted data included the first author, publication time and country, the sample size of each group, and the expected primary and secondary results.

We used the Cochrane bias risk assessment tool, which was recommended by the Cochrane manual, to assess the quality of methods included in the study. This tool performed bias risk assessment from six aspects: random allocation method, allocation concealment scheme, blind method, integrity of result data, selection report research results, and other biases. The author's judgment was divided into “low risk,” “high risk,” and “unclear risk” of bias.

### 2.4. Statistical Method

RevMan5.4 software was used for meta-analysis. Two-sided *P* < 0.05 indicates that the difference is statistically significant. The risk ratio (RR) and its 95% confidence interval (CI) were used to analyze the dichotomous variables. The heterogeneity test was conducted through *I*^2^. The fixed effect model was in the presence of no obvious interstudy heterogeneity as indicated by *P* > 0.05 and *I*^2^ < 50%. Otherwise, the random effect model was employed for significant interstudy heterogeneity. Subgroup and sensitivity analyses were used to explore the source of heterogeneity. The analysis result was presented by the forest map, and the publication bias was displayed by the funnel map and Egger's test.

## 3. Results

### 3.1. Literature Search Results

**Figure 1:**
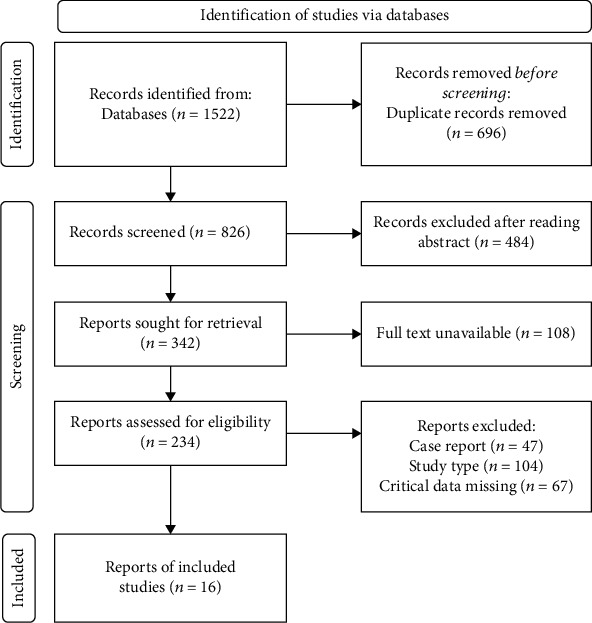
Flow chart of literature screening.

A total of 1522 English contributions were obtained through database retrieval, of which 826 were included after screening and eliminating duplicate literature. After reading the literature title and abstract, 16 studies [3–18] were finally included. The flow chart is shown in Figure 1.

### 3.2. Basic Information of the Included Studies

The included studies compared perineal massage versus no perineal massage during prenatal care. All included studies were conducted on pregnant women or their partners at 34-36 weeks of gestation. The included studies were reported from Asia, Europe, North America, Africa, and Oceania. Four studies were from Egypt [3–6], two from Canada [7, 8], and one from Australia [9], Japan [10], Ireland [11], Spain [12], Austria [13], Turkey [14], Iran [15], Nigeria [16], the UK [17], and the United States [18], respectively. The largest sample size was reported from Australia [9], with 1340 cases. A total of 6487 patients were included in the sample, including 3211 in the intervention group and 3267 in the control group. The basic characteristics of the literature and the assessment of risk of bias were shown in Table 1.

### 3.3. Perineal Tear

**Figure 2:**
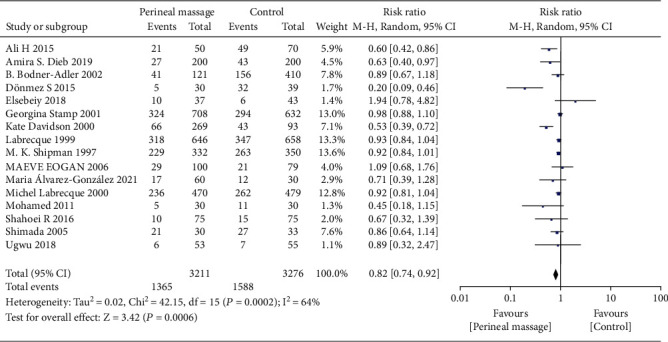
Forest map: effect of prenatal perineal massage on perineal tear.

**Figure 3:**
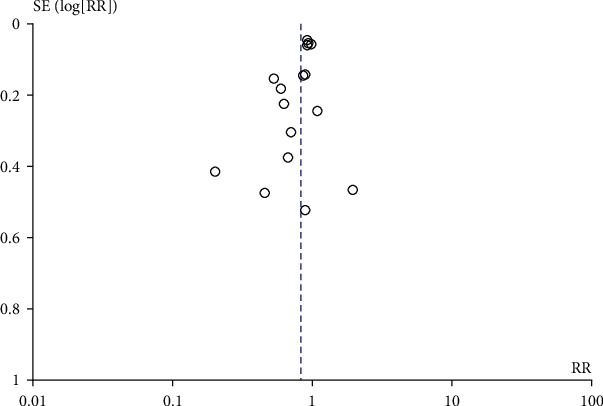
Funnel diagram: effect of prenatal perineal massage on perineal tear.

**Figure 4:**
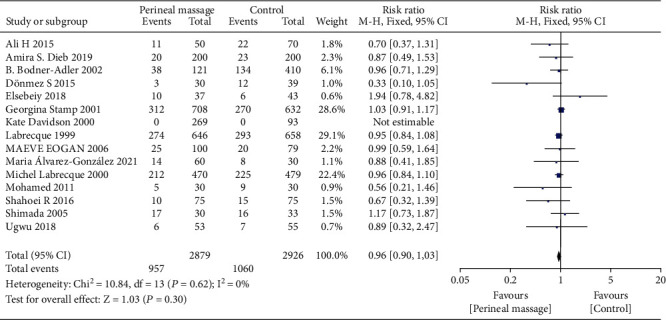
Forest map: effect of prenatal perineal massage on 1-2 degree perineal tear.

**Figure 5:**
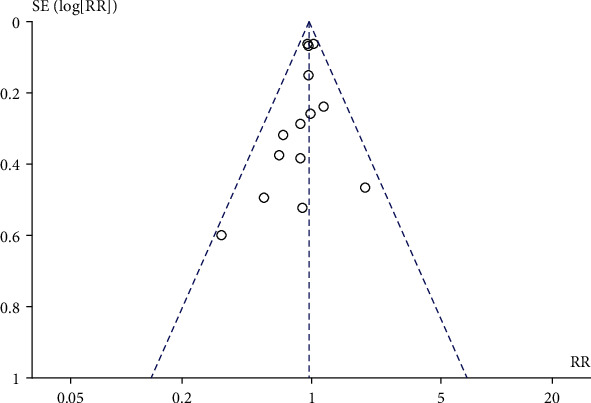
Funnel diagram: effect of prenatal perineal massage on 1-2 degree perineal tear.

**Figure 6:**
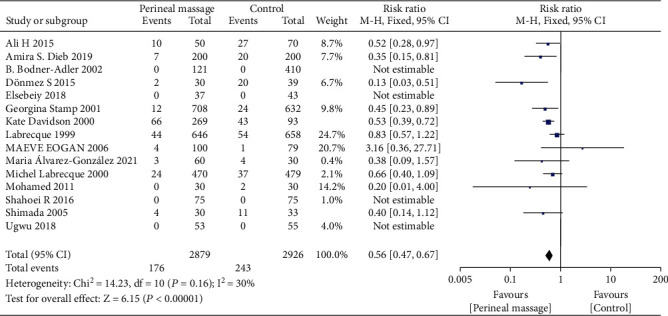
Forest map: effect of prenatal perineal massage on 3-4 degree perineal tear.

**Figure 7:**
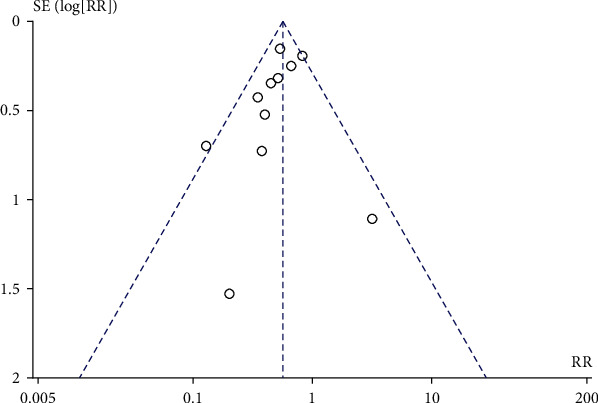
Funnel diagram: effect of prenatal perineal massage on 3-4 degree perineal tear.

A total of 16 literature compared the effect of prenatal perineal massage on the perineal tear. Significant interstudy heterogeneity (Chi^2^ = 42.15, *P* = 0.0002, *I*^2^ = 64%) was noted, for which the random effect model was used. Compared with the control group, prenatal perineal massage reduced the risk of perineal tear (RR = 0.82, 95% CI [0.74-0.92], *P* < 0.001) (Figure 2). The funnel plot and Egger's test showed that the scatter points were roughly symmetrically distributed, with no publication bias (*P* > 0.05) (Figure 3). To explore the source of heterogeneity, subgroup analysis was carried out according to the degree of perineal tear. There was no significant difference between the intervention group and the control group (RR = 0.96, 95% CI [0.90, 1.03], *P* = 0.30), and there was no heterogeneity between the studies (Chi^2^ = 10.84, *P* = 0.62, *I*^2^ = 0%) (Figure 4). The funnel plot and Egger test showed that the scatter points were biased to the left, and there was publication bias (*P* > 0.05) (Figure 5). Compared with the control group, prenatal perineal massage significantly reduced the incidence of 3-4 degree tear of perineum (RR = 0.56, 95% CI [0.47, 0.67], *P* < 0.00001), and there was no heterogeneity among the studies (Chi^2^ = 14.23, *P* = 0.16, *I*^2^ = 30%) (Figure 6). The funnel plot and Egger test showed that the scatter points were biased to the left, and there was publication bias (*P* > 0.05) (Figure 7).

### 3.4. Lateral Episiotomy

**Figure 8:**
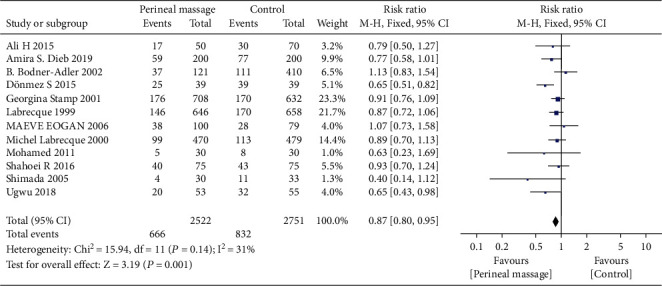
Forest map: effect of prenatal perineal massage on lateral episiotomy.

**Figure 9:**
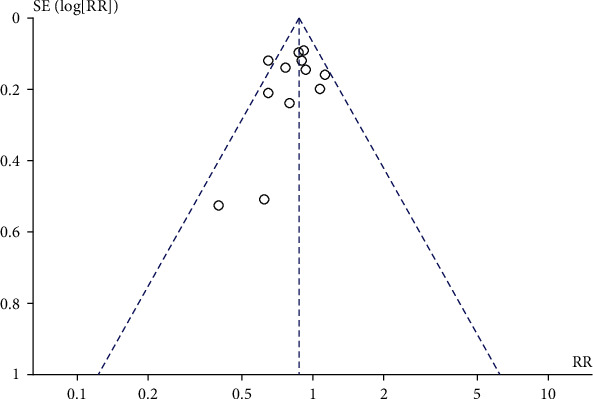
Funnel diagram: effect of prenatal perineal massage on lateral episiotomy.

Compared with the control group, prenatal perineal massage reduced the risk of lateral episiotomy (RR = 0.87, 95% CI [0.80, 0.95], *P* = 0.001), and the heterogeneity test result was *P* = 0.14, *I*^2^ = 31% (Figure 8). There was no heterogeneity among the studies. The funnel plot and Egger test showed that the scatter points were roughly symmetrical with no publication bias (*P* > 0.05) (Figure 9).

### 3.5. Natural Childbirth

**Figure 10:**
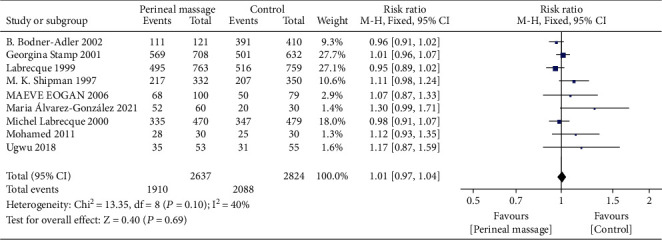
Forest map: effect of prenatal perineal massage on natural delivery.

**Figure 11:**
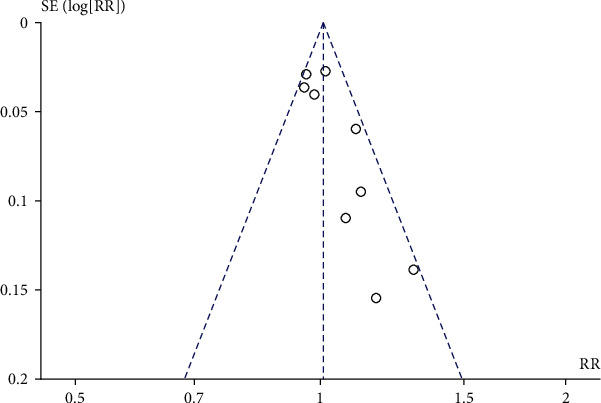
Funnel diagram: effect of prenatal perineal massage on natural delivery.

Compared with the control group, there was no significant difference in vaginal natural delivery in the prenatal perineal massage group (RR = 1.01, 95% CI [0.97~1.04], *P* = 0.69). There was no heterogeneity between studies (Chi^2^ = 13.35, *P* = 0.69, *I*^2^ = 40%) (Figure 10). The funnel plot and Egger test show that the scatter distribution is biased to the right, and there may be publication bias (*P* > 0.05) (Figure 11).

### 3.6. Perineal Pain

**Figure 12:**
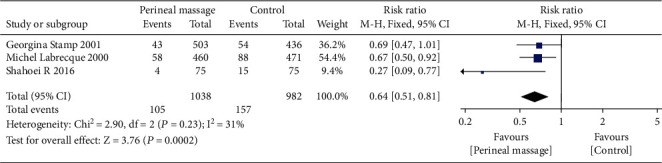
Forest map: effect of prenatal perineal massage on perineal pain 3 days after delivery.

**Figure 13:**
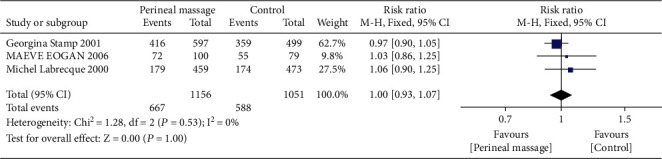
Forest map: effect of prenatal perineal massage on perineal pain 3 months postpartum.

We analyzed the perineal pain of parturients at 3 days and 3 months postpartum, respectively. The results showed that prenatal perineal massage reduced the pain risk of parturients at 3 months postpartum (RR = 0.64, 95% CI [0.51, 0.81], *P* = 0.0002) than the control group. There was no significant heterogeneity among the studies (Chi^2^ = 2.90, *P* = 0.23, *I*^2^ = 31%) (Figure 12). Egger's test showed that there was no publication bias among the literatures (*P* > 0.05). There was no significant difference in perineal pain between the intervention group and the control group at 3 days postpartum (RR = 1.00, 95% CI [0.93, 1.07], *P* = 1.00), and there was no significant heterogeneity among the studies (Chi^2^ = 1.28, *P* = 0.53, *I*^2^ = 0%) (Figure 13). Egger's test showed that there was no publication bias among the literatures (*P* > 0.05).

### 3.7. Urinary Incontinence

**Figure 14:**
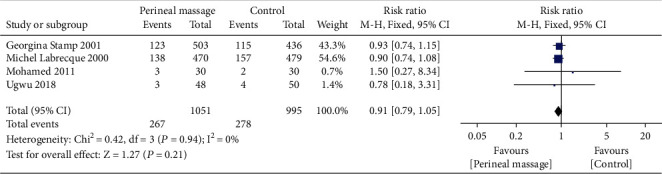
Forest map: effect of prenatal perineal massage on postpartum urinary incontinence.

Compared with the control group, there was no significant difference in urinary incontinence at 3 months postpartum in the prenatal perineal massage group (RR = 0.91, 95% CI [0.79~1.05], *P* = 0.21). There was no heterogeneity between studies (*P* = 0.94, *I*^2^ = 0%) (Figure 14). Egger's test showed that there was no publication bias among the literatures (*P* > 0.05).

### 3.8. Fecal Incontinence

**Figure 15:**
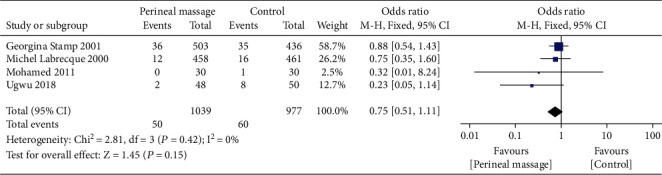
Forest map: effect of prenatal perineal massage on postpartum fecal incontinence.

Compared with the control group, there was no significant difference in fecal incontinence at 3 months postpartum in the prenatal perineal massage group (RR = 0.75, 95% CI [0.51~1.11], *P* = 0.15) (Figure 15). There was no heterogeneity between studies (*P* = 0.42, *I*^2^ = 0%) (Figure 15). Egger's test showed that there was no publication bias among the literatures (*P* > 0.05).

## 4. Discussion

Although perineal injury, a common complication of vaginal delivery, is not life-threatening to both the mother, its associated symptoms such as perineal pain, urinary incontinence, fecal incontinence, and difficulty in sexual intercourse seriously affect the patient's physical and mental health [19]. In this meta-analysis, the authors found that prenatal perineal massage significantly reduced the incidence of perineal tears and episiotomy, especially for 3^rd^-4^th^ degrees of perineal tears. In addition, prenatal perineal massage could significantly reduce the incidence of perineal pain 3 months after delivery. There was no significant difference in terms of incidence of vaginal delivery, perineal pain, urinary incontinence, and fecal incontinence between the prenatal perineal massage group and the control group.

Our study result is consistent with the previous studies [2, 20] that demonstrated that prenatal perineal massage can reduce the incidence of perineal tear and perineal incision. Furthermore, our study demonstrated the beneficial effect of prenatal perineal massage in reducing the risk of third- and fourth-degree perineal tears, which is consistent with that reported by Mohamed et al. [20]. However, in the systematic review of 2008 [21] and 2013 [2] by Beckmann et al., there was no difference in different degrees of perineal tear rate between prenatal perineal massage and the control group. This disparity might be explained by the fact that the study by Beckmann et al. only included 4 studies with a total of 2497 pregnant women, which is obviously much smaller in sample size as compared with the present study. Perineum incision during delivery is also a common cause of perineum injury. Our study showed that prenatal perineum massage could reduce the risk of perineum incision during delivery as compared with the control group, which is consistent with the results by Mohamed et al. and Beckmann et al. Moreover, Aquino et al. [22] even found that perineum massage during delivery could reduce the risk of perineum incision. Theoretically, perineal massage can stimulate skin nerve endings, promote tissue blood circulation, improve the elasticity and ductility of perineal tissue, reduce perineal incision, and improve perineal tear.

Perineal injury during childbirth leads to different complications for women, such as perineal pain. Then, we found that perineal massage could reduce the incidence of perineal pain at 3 months postpartum as compared with the control group. There was no significant difference in the incidence of perineal pain at 3 days postpartum. This is consistent with the results of Beckmann Michael and Stock Owen [2]. The decrease in perineal pain at 3 months postpartum may be related to the fact that prenatal perineal massage can reduce the incidence of perineal injury and perineal incision.

However, there were no significant differences between the two groups with regard to other secondary outcomes, such as the risk of urinary or fecal incontinence at 3 months postpartum and the incidence of spontaneous vaginal delivery. It may be due to the long follow-up time and women's self-esteem. Thus, the follow-up of urinary incontinence or fecal incontinence was difficult, and the data were incomplete. Of the 16 experiments in this study, only 4 reported urinary incontinence or fecal incontinence 3 months after delivery, and the number of samples was relatively small. In the study by Mohamed et al. [20] that analyzed only 3 experiments, prenatal perineal massage reduced the risk of anal incontinence (including fecal incontinence and gas incontinence) but did not reduce the risk of urinary incontinence. Given the relatively small sample size, we believe that additional investigations are entailed to explore the effect of perineal massage on urinary/fecal incontinence.

Reducing perineal injury caused by childbirth is pivotal for enhancing women's physical and mental health [19, 23]. According to our research, prenatal perineal massage can reduce the risk of perineal tear, especially the risk of 3^rd^ -4^th^ degree perineal tear. It can also reduce the risk of perineal incision during delivery and perineal pain 3 months after delivery. Previous studies have confirmed that prenatal perineal massage could benefit pregnant women [2, 8, 20]. However, factors like maternal self-esteem, obesity, and inconvenience make implementation of prenatal perineal massage difficult [24]. Studies have shown that [25] the application of smartphone Apps can better help pregnant women master and apply this helpful technology and enable pregnant women to adhere to the use of prenatal perineal massage from the 34th week of pregnancy to delivery. Obstetrics and gynecology medical staff can learn from this method, publicize and popularize this technology, and encourage and recommend pregnant women to have a prenatal perineal massage before 34 weeks of pregnancy.

The main advantages of this meta-analysis are based on clear definition, strict inclusion and exclusion criteria, a comprehensive retrieval strategy, and a large sample size. According to the retrieval, our research is the most and latest sample in this field. Our limitation is the relatively limited observation indicators included. For example, the effect of prenatal perineum massage on improving postpartum sexual satisfaction and the risk of urinary incontinence and fecal incontinence at 3 months after delivery needs to be further confirmed. These outcomes are directly related to the quality of life of patients and their families that entail continued investigations.

## 5. Conclusion

Antenatal perineal massage reduces the risk of perineal tears (especially 3^rd^-4^th^ degree) during vaginal delivery, episiotomy, and perineal pain 3 months postpartum. Therefore, obstetrics and gynecology professionals should consider popularizing prenatal perineal massage.

## Figures and Tables

**Table 1:** Basic characteristics of the literature and assessment of risk of bias.

Author	Country	Year	No. of patients		Risk of basis
			Perineal massage	Control	
Ali H	Egypt	2015	50	70	High
Amira S. Dieb	Egypt	2019	200	200	High
B. Bodner-Adler	Austria	2002	121	410	Uncertain
Dönmez S	Turkey	2015	30	39	High
Elsebeiy	Egypt	2018	37	43	Low
Georgina Stamp	Australia	2001	708	632	Uncertain
Kate Davidson	United States	2000	269	93	Uncertain
Labrecque	Canada	1999	646	658	Uncertain
M. K. Shipman	UK	1997	332	350	Low
Maeve Eogan	Ireland	2006	100	79	High
María Álvarez-González	Spain	2021	60	30	Uncertain
Michel Labrecque	Canada	2000	470	479	Uncertain
Mohamed	Egypt	2011	30	30	Uncertain
Shahoei R	Iran	2016	75	75	Uncertain
Shimada	Japan	2005	30	33	Low
Ugwu	Nigeria	2018	53	55	High

## Data Availability

The data used to support the findings of this study are included within the article.
